# What has driven the spatial spillover of China’s out-of-pocket payments?

**DOI:** 10.1186/s12913-019-4451-0

**Published:** 2019-08-30

**Authors:** Ruijie Zhang, Jinghua Li, Xiaochun Du, Tianjiao Ma, Li Zhang, Qian Zhang, Fang Xia

**Affiliations:** 1 School of Public Health, Jilin University, Changchun City, Jilin Province China; 2 School of Management, Changchun University of Chinese Medicine, No. 1035, Bo Shuo Road, Jing Yue District, Changchun City, 130117 Jilin Province China

**Keywords:** Out-of-pocket payments, Spatial cluster, Spatial Durbin model, Spatial spillover sources

## Abstract

**Background:**

Even though China launched a series of measures to alleviate several financial burdens (including health insurance scheme, increased government investment, and so on), the economic burden of health expenditure has still not been alleviated. Out-of-pocket payments (OPPs) show not only a time correlation but also some degree of spatial correlation. The aims of the current study were thus to identify the spatial cluster of OPPs, to investigate the main factors affecting variation, and to explore the spatial spillover sources of China’s OPP.

**Methods:**

Global and local spatial autocorrelation tests were validated to identify the spatial cluster of OPPs using the panel data of 31 provinces in China from 2005 to 2016. The Spatial Durbin Model, established in this paper, measured the spatial spillover effect of OPPs and analyzed the possible spillover sources (demand, supply, and socio-economic factors.

**Results:**

OPPs were found to have a significant and positive spatial correlation. The results of the Spatial Durbin Model showed the direct and indirect effects of demand, supply, and socio- economic factors on China’s OPPs. Among the demand factors, the direct and indirect correlation (elasticity) coefficients were positive. Among the supply factors, the direct and indirect effects of the share of primary health beds on residents’ OPPs were negative. The ratio of health technicians in hospitals to those in primary health institutions on per capital OPPs had a significant indirect effect. Among the socio-economic factors, the direct effects of GDP, government health expenditure, and urbanization on OPPs were found to be positive. There were no significant indirect effects of socio-economic factors on OPPs.

**Conclusion:**

This paper finds that China’s OPPs are not randomly distributed but, overall, present a positive spatial cluster, even though a series of measures have been launched to promote health equity. Socio-economic factors and those associated with demand were found to be the main influences of variation in OPPs, while demand was seen to be the driver of the positive spatial spillover of OPPs, whereby effective supply could inhibit these positive spillover effects.

**Electronic supplementary material:**

The online version of this article (10.1186/s12913-019-4451-0) contains supplementary material, which is available to authorized users.

## Background

In the healthcare financing sector, out-of-pocket payments (OPPs) are the direct outlay of cash that the patient or the family pays to the healthcare provider. OPPs are considered to be a more objective index by which to reflect inequitable health care services and inequity in terms of the financial burden [1, 2]. Extremely high OPPs relative to income may result in financial catastrophe and health inequity [3]. As an essential health financing resource, rational OPPs can improve health equity by ensuring the fair allocation and effective use of health resources.

**Fig. 1:**
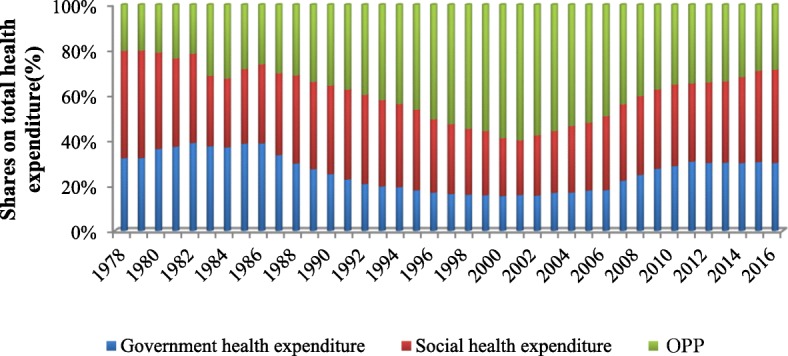
The shares on total health expenditure from 1978 to2016 in China

**Fig. 2:**
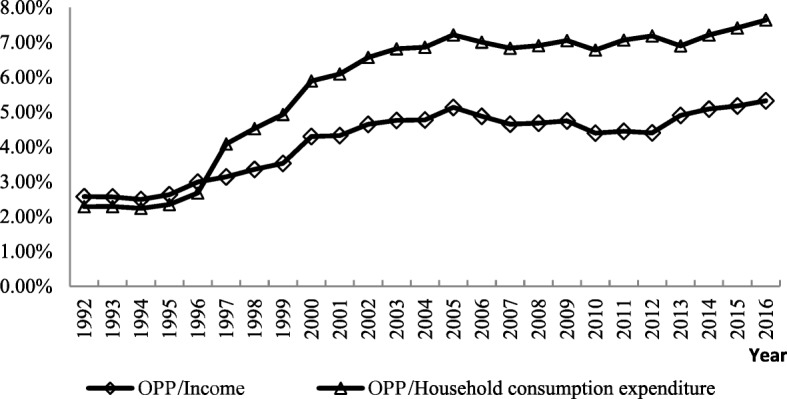
The proportion of health expenditure to disposable income and household consumption expenditure from 1992 to 2016 in China

After the Chinese economic reform in 1978, the government dismantled the previous publicly funded healthcare system, which led to a rapid increase in OPPs, with their share in total health expenditure (THE) rising from 20% in 1978 to nearly 60% in 2002. As a result, the government launched a series of measures to alleviate financial burdens(through, for instance, a social health insurance scheme, increasing government investment in health care, and so on)reducing the share of OPPs in THE to 28.78% in 2016. However, the economic burden of health expenditure has still not been alleviated (see Figs. 1 and 2). As Qian Long [4] has argued, the rapid increase of public funding in China, as part of the reform strategy, did not mitigate OPPs for healthcare from 2003 to 2013. China’s OPPs are still relatively high and the overall incidence of catastrophic health expenditure (CHE) is about 13% [5, 6].

While the health financing sources are the same across provinces in China, including government health expenditure (GHE), social health expenditure(SHE), and OPPs, the proportion of different sources of financing differs from province to province. In 2016, the central region, which has an average income in comparison to the other regions, had the highest share of OPPs in THE, accounting for more than 30% which means that there remains a high risk of catastrophic health expenditure [7]. The eastern region has the highest income among the regions, as well as the highest SHE (47.22%), while the poorer western provinces have the highest proportion of GHE (38.50%) (see Additional file 1: Table S1).

Health consumption demonstrates a high reliance on health resources supplements, while health institutions are the foundation of other health resources. The Chinese health institution system includes primary health institutions, which are responsible for prevention, medical care, and rehabilitation services in certain regions, as well as Level II and Level III hospitals, which are equipped to afford a higher level of medical services than primary health institutions. The level of health treatment and the prices of hospitals are higher than those of primary health institutions, and the Chinese government does not offer citizens a choice of medical institutions on their first diagnosis. As such, residents tend to select hospitals when they need healthcare services. The health institutions in developed areas are able to provide better health services than those in underdeveloped areas, which increases the possibility of residents seeking cross-regional healthcare services.

In order to pool the risks of health financing, the Chinese government built the New Rural Cooperative Medical Insurance (NCMI) in 2003, and, in 2007, developed the Urban Residents Medical Insurance (URMI) and improved the Urban Employees Medical Insurance (UEMI). Chinese social medical insurance is provincially managed; thus different provinces have different institutions and requirements. Developed provinces have a higher reimbursement ratio, and citizens in other provinces can be reimbursed in all insured places, so those residents who have medical insurance more likely to seek better medical services. Under the Chinese health institutions system and the health insurance system, it is of great significance to understand the spatial payment pattern and driving factors of OPPs for controlling out-of-pocket expenditure.

In view of a great deal of attention being paid to the equity of healthcare systems, healthcare expenditure (HCE) drivers have become a topical issue in academia. Many scholars have analyzed whether prior health expenditure can explain current health expenditure using the time series method [8–11]. Others have explored the determinants of healthcare expenditure using panel regression methods [12]. Previous studies have thus been based on time series data or panel data, whereby unobserved heterogeneity may stem from omitted common variables that affect geographic areas differently. Cross-provincial behaviors are not surprising, as one of the economic activities, though patients who are not local are not eligible for medical insurance reimbursement in other regions, meaning that OPPs can also correlate spatially with the neighboring regions [13]. If the spatial correlation of OPPs is ignored, policies and decisions made by public health policy-makers may favor the control of local OPPs and ignore the impact on other regions. Although previous studies have suggested that the level of economic development and the patient’s medical condition have a significant influence on OPPs, the latter may, arguably, be seen to have a geographical spillover effect given the influential factors of health [14]. These latent, common factors may induce cross-section dependence and lead to inconsistent estimation coefficients in regressions if hidden aspects are correlated with the explanatory variables. Overall, existing techniques focusing on time series and panel data can predict the tendency of OPPs and construct two-dimensional models, while spatial econometric models can adopt latent spatial correlation so as to explore regional variations in OPPs.

While literatures exploring the determinants of healthcare expenditure using panel or cross-section data indicates the sources of the variation of healthcare expenditure at a specific point in time or period, they hardly reflect the spatial effects and spillover sources of OPPs [15]. Human economic activities carried out in a certain time and space can show not only a temporal correlation but also some degree of spatial correlation. The purpose of the current study is to examine the spatial correlation of OPPs using a balanced panel dataset from 2005 to 2016, which has not been under taken in previous research. As mentioned earlier, the spatial correlation of OPPs can mirror regional inequities in the distribution of health resources and ineffective health resource utilization. In this research, we first tested the spatial correlation of OPPs, and then investigated the spatial spillover effect and the sources of OPP spillover in China, especially at the provincial level.

Grassetti [16] has demonstrated that, given residents’ demands for health services, the interregional imitative supply, and socio-economic factors, spatial interaction of contiguous provinces may lead to the clustering of residents’ health-seeking-behavior, which empirically explains the spatial correlation pattern of OPPs. In order to understand health inequity, the spatial effects of HCE need to be analyzed and introducing the spatial econometric model can solve the problem [17–20]. To the best of our knowledge, research pertaining to the spatial interactions of public health expenditure (PHE) across different jurisdictions has shown that a district tends to increase its own healthcare expenditure in response to the rise in the expenditure of its neighboring jurisdictions. For example, Chandra and Amitabh [18] have identified that PHE has a positively significant spatial correlation using the panel data of the United States for 2000–2009 years. Vincenzo [19] argues that the positive effect is also significant in Italy, but that the spillover effect under institutional constraints is close to zero. A few studies have verified the spatial spillover effect of OPPs and explored spillover sources. Matteo etal. [20] investigate the spatial dependence between OPPs, associated with basic treatment and complex medical procedures, using a spatial econometrics model. They found that a significant positive spillover effect occurs with basic treatment due to the diffusion of best practices, whereas this effect is not significant for complex treatment due to strict restrictions on health planning. These studies have laid the foundation for exploring the spatial aggregation and spillover effects of OPPs in China.

According to related research on the spatial effect of healthcare expenditure, spatial spillovers may arise from the heterogeneity of residents’ purchases and health conditions on the demand side, supply-side characteristics such as health resources and knowledge spillover, and the spatial interaction of socio-economic factors [16, 18–20].

The spatial spillover effect of OPPs may be induced by residents’ demands. Zhang and Ma [21, 22] have indicated that secondary or tertiary hospitals of higher quality are the main providers of health services, and that medical insurance has no significant impact on patients’ visits to primary care facilities, which implies that the quality of medical service is a key factor in residents’ choice of medical institutions. Therefore, they may seek better healthcare services by crossing borders when they recognize that their local health services cannot meet their demands. Lippi, Bruni, and Bose [20, 23] suggested that the indicator representing demand—the aged population in State A—has a significant, indirect effect on the health spending of neighboring states. This is because the elderly population from neighboring states may migrate and settle in State A in search of better and improved health facilities. However, the possibility of seeking high-quality services will decrease with an increase in geographical distance.

A rationale for this spatial spillover lies in the differences between health resources and knowledge spillover, on the supply side, among different areas. The availability and sophistication of technology have been found to exert the most pronounced effects on attracting patient inflows [24]. The improvement of medical technology in a region is more likely to meet residents’ demands and thus increase the market share of healthcare services [25]. Empirically, Bin Zhu’s [26] results indicate that spillover effects of the health labor market exist in China and suggested that, if government investment in the healthcare in one unit were to be relatively higher than in other units, health professionals from adjacent units would most likely be attracted to this unit through migration flows. Moreover, the interaction of knowledge among regions can lead to technology transfer, thus improving the quality of care through the imitation of medical technology [27]. According to recent studies, OPPs may also be driven by the externality and outlay of supply-side factors in neighboring areas in China.

Spatial spillover can be generated by fiscal competition or related interactions across regions. In an environment of economic competition, regional governments expect to promote regional economic development through the improvement of residents’ health and consumption [28]. GDP and urbanization level have been found to be positively associated with regional health expenditure [19, 23, 29]. A higher level of development in socioeconomic factors can attract more people to live and work in a particular area, which will, in turn, promote the spatial mobility of OPPs. Nevertheless, residents with urban employee basic medical insurance and with urban residents basic medical insurance are more likely to switch from lower-tier to higher-tier hospitals [21]. In other words, urban residents are more able and willing to seek out high-quality health services. From this, it can be inferred that the level of urbanization drives the spillover effects of OPPs in China. The government’s health expenditure could impact positively on healthcare resources in local and surrounding regions due to the competitive and mimetic effects of government fiscal spillover, which will influence the healthcare-seeking behaviors of residents from surrounding regions [30, 31]. However, whether socio-economic factors can explain the spatial spillover effect of OPPs remains to be explored in the context of China’s economic transformation.

There are two potential contributions of this study. On the one hand, it validates global and local spatial autocorrelation tests to identify the spatial clustering of OPPs using the panel data of 31 provinces in China from 2005 to 2016. On the other hand, the constructed spatial econometric model in this paper measures the spatial spillover effect of OPPs and analyzes the possible spillover sources (demand, supply, and socio-economic factors) from the perspective of health equity in resource distribution and healthcare utilization. The study aims to solve the problem that previous researchers have hardly reflected on the spatial patterns of OPPs, and to raise awareness of the bias or inefficiency estimation that can occur if spatial correlation is ignored.

## Methods

### Data

For the purposes of this study, we utilized a balanced set of provincial panel data concerning health spending patterns, covering a dependent variable(per capita OPPs)and nine independent variables (the level of aging, per capita income, mortality rate, the ratio of visits to hospitals versus those to primary health institutions, maternal mortality, the share of primary health beds, the ratio of health technicians in hospitals to those in primary health institutions, per capita GDP, government health expenditure, education levels, and urbanization levels) in 31 provinces during 2005–2016, yielding a total number of 372observations.The dataset was extracted from two sources: the *China Statistical Yearbook* and the *China Health Statistics Yearbook*. OPPs are defined as the direct medical costs borne by households. Referring to the literature studying the spatial effects of healthcare expenditure, we selected a series of explanatory variables, including three categories: demand, supply, and socio-economic factors. The demand-side factors include the level of aging, per capita income, mortality rate, and the ratio of visits to hospitals and those to primary institutions. Maternal mortality, the share of primary health beds, and the ratio of health technicians in hospitals and those in primary institutions were used to represent the technical, material, and human resources supplied by China’s health service system. We used per capita GDP, government health expenditure, education level, and urbanization level to reflect the socio-economic situation. Per capita OPPs, the level of aging, per capita income, and all of the socio-economic factors were exacted from *China Statistical Yearbooks*, and other factors were collected from *China Health Statistics Yearbooks* during 2006–2017.

Per capita OPPs, GDP, and income level were converted into real values according to the province-specific consumer price index obtained from the *China Statistical Yearbook*, which took the form of natural logarithms in the process of constructing the econometric model. All of the variables used and their basic descriptive statistics are summarized and presented in Table 1. The first and second columns report the means and standard deviations. We used Opengeoda1.2.0 and Stata13.1 software to deal with the database.

**Table 1:** Descriptive statistics of variables

Variable	Mean	Standard deviation	Min	Max
Logged per capita OPP (RMB)	6.451	0.615	4.496	7.902
Logged per capita income (RMB)	2.970	0.668	1.287	4.731
Aging level (%)	9.100	1.800	4.800	14.400
Mortality rate (%)	1.776	0.124	1.437	1.985
Ratio of visits in hospitals and those in primary institutions	1.368	1.032	0.323	7.656
Share of primary health care beds (%)	20.10	11.00	3.700	76.30
Ratio of health technicians in hospitals and those in primary institutions	5.896	22.164	0.852	362.333
Maternal mortality rate (%)	2.963	0.840	0.182	5.671
Logged gross Domestic Product (RMB)	1.765	0.415	0.694	2.581
Logged education level (Year)	2.100	0.175	1.319	2.487
Logged government health expenditure (RMB)	5.840	0.866	3.654	7.570
Urbanization level (%)	50.90	14.80	20.70	89.60
Observations	372			

Notes: 1 Per capita OPP, Per capita income, GDP, Education level and Government health expenditure take natural logarithm forms

2 Aging level = population older than age 65/total population;

3 Ratio of visits in hospitals and those in primary institutions = the number of visits in hospitals/those in primary health institutions;

4 Share of primary health care beds = the number of health beds in primary health institutions/all of health beds;

5 Ratio of health technicians in hospitals and those in primary institutions = the number of health technicians in hospitals/those in primary health institutions;

6 Urbanization level = urban population/total population

### Empirical methods

#### Spatial weight matrix

The premise of spatial econometric analysis is to measure the spatial distance between two regions, with spatial contiguity being a common distance measurement to define two spatial units [32]. There are three ways to determine the adjacency relationship: rook contiguity (two regions share a common border), bishop contiguity (two regions share a common vertex, but no common edge), and queen contiguity (two regions share a common border or vertex) [33].

This paper constructed a spatial weight matrix W_ij_ between Province i and Province j according to the principle of queen contiguity because the weight matrix was deemed more conducive to showing the surrounding correlation of spatial units than rook and bishop contiguity; the residents’ healthcare-seeking behaviors are not restricted by the boundary or the vertex [33]. W_ij_ is defined as follows:

$$ {\mathrm{W}}_{\mathrm{ij}}=\left\{\begin{array}{l}1\left(\mathrm{if}\kern0.17em \mathrm{provinces}\;\mathrm{i}\;\mathrm{and}\;\mathrm{j}\;\mathrm{share}\kern0.17em \mathrm{border}\kern0.17em \mathrm{or}\kern0.17em \mathrm{vertex}\right)\\ {}\kern3.839998em 0\left(\mathrm{otherwise}\right)\end{array}\right. $$

#### Spatial autocorrelation tests

The first step of the spatial econometric analysis was to test the spatial autocorrelation of the dependent variable using the Moran’s I index of the Exploratory Spatial Data Analysis (ESDA) method. If spatial autocorrelation were to be found, the spatial econometric model could then be established [34]. ESDA was divided into a global spatial autocorrelation analysis, to describe the spatial autocorrelation strength of China’s entire OPPs, and a local spatial autocorrelation analysis to examine whether there existed such a spatial autocorrelation of per capita OPP among neighboring provinces. Generally, the global Moran’s I was used to verify global autocorrelations, while the distribution maps of local indicators of spatial associations (LISA) can more intuitively describe key per capita OPPs’ clusters [35]. The global Moran’s I statistic for spatial autocorrelation strength is represented as follows:

1 $$ {\mathrm{Moran}}^{\prime}\mathrm{s}\ \mathrm{I}=\frac{\mathrm{n}{\sum}_{\mathrm{i}}{\sum}_{\mathrm{j}}{\mathrm{W}}_{\mathrm{i}\mathrm{j}}\left({\mathrm{y}}_{\mathrm{i}}-\overline{\mathrm{y}}\right)\left({\mathrm{y}}_{\mathrm{j}}-\overline{\mathrm{y}}\right)}{\sum_{\mathrm{i}}{\sum}_{\mathrm{j}}{\mathrm{W}}_{\mathrm{i}\mathrm{j}}{\sum}_{\mathrm{i}}{\left({\mathrm{y}}_{\mathrm{i}}-\overline{\mathrm{y}}\right)}^2}=\frac{\mathrm{n}{\sum}_{\mathrm{i}}{\sum}_{\mathrm{j}}{\mathrm{W}}_{\mathrm{i}\mathrm{j}}\left({\mathrm{y}}_{\mathrm{i}}-\overline{\mathrm{y}}\right)\left({\mathrm{y}}_{\mathrm{j}}-\overline{\mathrm{y}}\right)}{{\mathrm{S}}^2{\sum}_{\mathrm{i}}{\sum}_{\mathrm{j}}{\mathrm{W}}_{\mathrm{i}\mathrm{j}}} $$

where y_i_ and y_j_ represent per capita OPPs in Region i and Region j, *n* is the total number of regions, $\overline{\mathrm{y}}$ is the mean of observations, S^2^ is the variance of the observation variable, and W_ij_ is the element in the spatial weights matrix. The Moran’s I value lies in the range of [− 1,1]. A Moran’s I greater than zero indicates that there exists a positive spatial autocorrelation among the observations, while a Moran’s I lower than zero means that there is a negative spatial correlation between them. When Moran’s I is equal to zero, t here is no spatial autocorrelation among the observations.

The LISA statistic for spatial autocorrelation strength is represented as follows:

2 $$ \mathrm{LISA}=\frac{\left({\mathrm{y}}_{\mathrm{i}}-\overline{\mathrm{y}}\right)}{{\mathrm{S}}^2}{\sum}_{\mathrm{j}}{\mathrm{W}}_{\mathrm{i}\mathrm{j}}\left({\mathrm{y}}_{\mathrm{j}}-\overline{\mathrm{y}}\right) $$

where y_i_, y_j_, W_ij_ and S^2^ are the same as those used for the calculation of the Moran’s I index. A positive LISA value indicates that there are similar spatial cluster patterns (e.g., high-high or low-low) among the adjacent space regions. A negative LISA value indicates that there are different patterns (e.g., high-low or low-high) among these regions.

Standardized Z statistics can test whether the global or local spatial correlation is statistically significant; the Z formula is:

3 $$ \mathrm{Z}=\frac{\mathrm{I}-\mathrm{E}\left(\mathrm{I}\right)}{\sqrt{\mathrm{Var}\left(\mathrm{I}\right)}} $$

A significant and positive Z value indicates a positive spatial correlation; that is to say, similar observation values (high values or low values) tend to indicates spatial agglomeration. Nevertheless, a negative and significant Z represents a negative spatial correlation. When Z equals 0, this means that the observed values are randomly distributed.

#### Spatial Durbin model

The Spatial Durbin Model (SDM)—an extended combination of the spatial autocorrelation model and the spatial error model—can be used to consider the spatial lagged explanatory variables and interpret variables at the same time as capturing the spatial spillover of OPPs and exploring the sources of spatial spillover. The influence of the independent variables on per capita OPPs can be divided into direct effects and indirect effects. Direct effects measure the impacts of the independent variables in a particular region on local per capita OPPs. Indirect effects measure the average impacts of neighboring changes of a certain variable on local OPP. The expression is as follows:

4 $$ \mathrm{Ln}\ {\mathrm{Y}}_{\mathrm{i}.\mathrm{t}}=\mathrm{c}+\uprho {\mathrm{W}}_{\mathrm{i}\mathrm{j}}{\mathrm{LnY}}_{\mathrm{j}.\mathrm{t}}+\upbeta {\mathrm{X}}_{\mathrm{i},\mathrm{t}}+\uptheta {\mathrm{W}}_{\mathrm{i}\mathrm{j}}{\mathrm{X}}_{\mathrm{i}.\mathrm{t}}+{\uplambda}_{\mathrm{t}}+{\upmu}_{\mathrm{t}}+{\upepsilon}_{\mathrm{i},\mathrm{t}} $$

Where Y stands for per capitaOPPs.cis constant .i and t represent the province and year, respectively, j represents other provinces. X_i, t_ stands for a series of independent variables, β is the coefficient of the independent variables, and ρ is the current spatial autoregressive coefficient. θ denotes the regression coefficient of the spatial lag independent variables, ϵ is the error term, and λ_t_ and μ_t_ are the vector and period of the spatial fixed or random effects, respectively.

Referring to the method proposed by Elhorst (2010), the SDM can be transferred as the following form:

5 $$ {\mathrm{Y}}_{\mathrm{i}.\mathrm{t}}={\left(\mathrm{I}-\uprho \mathrm{W}\right)}^{-1}\left(\upbeta {\mathrm{X}}_{\mathrm{i},\mathrm{t}}+\uptheta {\mathrm{W}}_{\mathrm{i}\mathrm{j}}{\mathrm{X}}_{\mathrm{i}.\mathrm{t}}+{\uplambda}_{\mathrm{t}}+{\upmu}_{\mathrm{t}}+{\upepsilon}_{\mathrm{i},\mathrm{t}}\right) $$

Where I is an N*1 unit matrix, N is the number of cross-sections. The spatial Leontief inverse matrix can be spread as follows:

6 $$ {\left(\mathrm{I}-\uprho \mathrm{W}\right)}^{-1}=\mathrm{I}\ \left(\upbeta {\mathrm{X}}_{\mathrm{i},\mathrm{t}}+\uptheta {\mathrm{W}}_{\mathrm{i}\mathrm{j}}{\mathrm{X}}_{\mathrm{i}.\mathrm{t}}+{\uplambda}_{\mathrm{t}}+{\upmu}_{\mathrm{t}}+{\upepsilon}_{\mathrm{i},\mathrm{t}}\right) $$

Eq. (5) stands for the direct effect, while the remaining part is the indirect effect. The first partial derivative of the dependent variable to the independent ones is as follows:

7 $$ \frac{\updelta {\mathrm{Y}}_{\mathrm{i}}}{\updelta {\mathrm{X}}_{\mathrm{i}\mathrm{r}}}={\mathrm{S}}_{\mathrm{r}}{\left(\mathrm{W}\right)}_{\mathrm{i}\mathrm{i}}\ \mathrm{for}\ \mathrm{all}\ \mathrm{i}\ \mathrm{and}\ \mathrm{r} $$

8 $$ \frac{\updelta {\mathrm{Y}}_{\mathrm{i}}}{\updelta {\mathrm{X}}_{\mathrm{jr}}}={\mathrm{S}}_{\mathrm{r}}{\left(\mathrm{W}\right)}_{\mathrm{i}\mathrm{j}}\ \mathrm{for}\ \mathrm{all}\ \mathrm{i}\ne \mathrm{j}\ \mathrm{and}\ \mathrm{for}\ \mathrm{all}\ \mathrm{r} $$

9 $$ {\mathrm{S}}_{\mathrm{r}}\left(\mathrm{W}\right)={\left({\mathrm{I}}_{\mathrm{N}}-\uprho \mathrm{W}\right)}^{-1}\left({\mathrm{I}}_{\mathrm{N}}{\upbeta}_{\mathrm{r}}-{\upomega}_{\mathrm{r}}\mathrm{W}\right) $$

Where β_r_ represents the coefficient for the r_th_ independent variable, ω_r_ denotes the coefficient of the spatial lag term of the r_th_ independent variable, S_r_(W)_ii_ s the element in the diagonal line, indicating the impact of the independent variable in the i_th_ region on the dependent variable in the i_th_ region, which refers to the direct effect. The simple average of the elements in the diagonal line is the average direct effect. The off-diagonal elements, which reflect the impact of the independent variable of the j_th_ region on the dependent variable of the i_th_ region, refer to the indirect effect or the spillover effect. The simple average of all the off-diagonal elements is the average indirect effect. The summation of the average direct and indirect effect is the average total effect, which is also the average of all the elements.

According to the purpose of the study and the Hausman test, we finally chose the individual fixed effect-SDM.

## Results

### Geographical distribution of OPPs

**Fig. 3:**
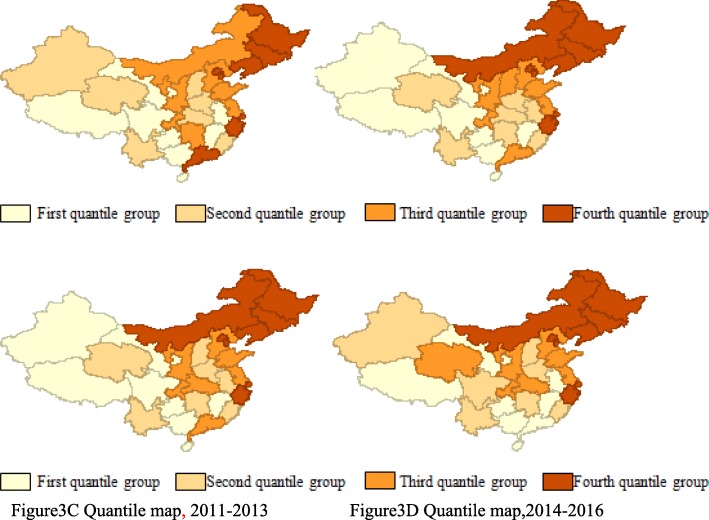
Quantile maps of OPPs from 2005 to 2016 in China

Figure 3 presents the geographical distribution of per capita OPPs in China during 2005–2016, with the darker color indicating higher per capita OPPs. From this, it can be seen that the northeastern OPPs (in Heilongjiang, Harbin, Jilin, and Inner Mongolia) are in the fourth quantile group with the highest level of per capita OPPs, while western per capita OPPs mostly occur in the first or second quantile group. The figure provides evidence of the positive spatial clustering of per capita OPPs in China. Therefore, it can be speculated that China’s per capita OPPs may have a positive spatial correlation rather than a random distribution. If there, indeed, exists a spatial correlation of per capita OPPs, this makes it possible to investigate the spatial spillover effect and sources of this expense using a spatial econometric model, especially at the provincial level.

### Spatial autocorrelation tests

The global spatial correlation test shows the spatial correlation strengthening of China’s per capita OPPs. The latter was found to have a significant and positive spatial correlation under the 5% significant level, according to the results of the global Moran’s I Test (as presented in Table 2). Specifically, the level of spatial correlation strengthening first increased and then decreased, taking 2012 as the demarcation of this change.

**Table 2:** Global Moran’s I test of OPP in China, 2005–2016

Year	Moran’s I	SD	Z	P
2005	0.244	0.093	3.051	0.03
2006	0.297	0.109	2.983	0.03
2007	0.368	0.107	3.755	0.01
2008	0.36	0.099	4.025	0.01
2009	0.431	0.109	4.298	0.01
2010	0.436	0.088	5.352	0.01
2011	0.43	0.111	4.061	0.01
2012	0.457	0.105	4.833	0.01
2013	0.419	0.099	4.779	0.01
2014	0.324	0.098	3.597	0.01
2015	0.323	0.106	3.565	0.01
2016	0.263	0.094	3.324	0.01

**Fig. 4:**
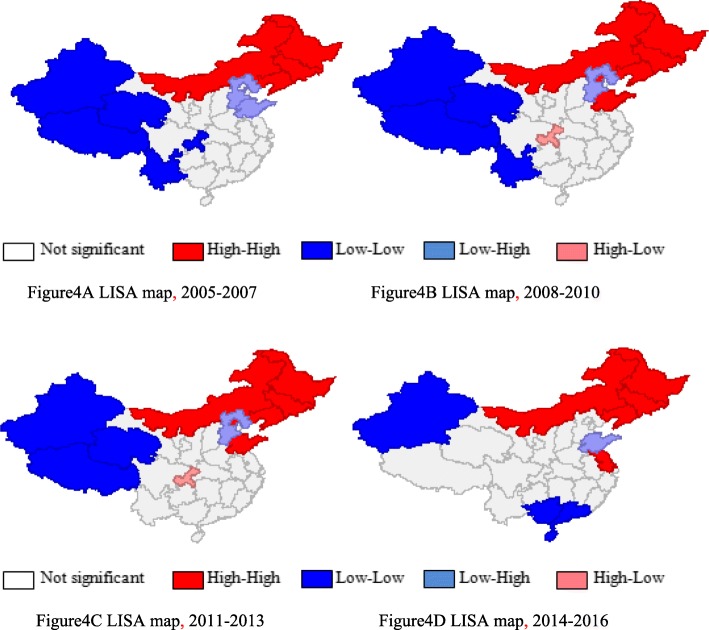
LISA distribution maps from 2005 to 2016 in China

In order to explore the clustering pattern of OPPs visually in local areas, a local spatial association test was carried out. As reported in the LISA distribution maps in Fig. 4, the OPPs in adjacent provinces had clear local correlation. Here, the significant LISA indices are mainly distributed in the high-high group and low-low group, while provinces in the low-high group or high-low group show less of this distribution. Specifically, the northeastern and western per capita OPPs are located in the high-high group and low-low group, respectively, while the north and central per capita OPPs are in the non-significant group.

### Overall results of SDM

Table 3 presents the direct and indirect effects of the SDM. The R-square value (0.963), reported by the spatial Durbin fixed-effect model, can explain 96.13% of the OPPs’ variation, indicating that this model is appropriate for this paper. The spatial correlation coefficient is 0.453 (at the 1% significance level), meaning that there is a significant positive spatial correlation among residents’ OPPs; namely, one region will increase their OPPs by 0.453% when the per capita OPPs of a neighboring region increases by5%. The spatial correlation of per capita OPPs among these provinces reveals the inequity of the distribution of health resources and the utilization of health services in China.

**Table 3:** The direct and indirect effects of the spatial Durbin model

Variable	Direct effect	Indirect effect	Total effect
Logged per capita income (RMB)	0.398***	0.206*	0.604***
	(0.086)	(0.130)	(0.109)
Aging level (%)	0.127*	0.616***	0.743
	(−0.879)	(1.652)	(1.600)
Mortality rate (%)	0.156*	0.353*	0.509**
	(0.107)	(0.217)	(0.24)
Ratio of visits in hospitals and those in primary institutions	0.013*	0.034**	0.047*
	(0.010)	(0.015)	(0.015)
Share of primary health care beds (%)	−0.09**	−0.401***	−0.491***
	(0.049)	(0.100)	(0.094)
Ratio of health technicians in hospitals and those in primary institutions	0.020	−0.014***	0.006**
	(0.022)	(0.056)	(0.058)
Maternal mortality rate (%)	−0.014	−0.060	− 0.074
	(0.020)	(0.222)	(−0.074)
Logged gross Domestic Product (RMB)	0.050	−0.092	−0.042
	(0.08)	(0.155)	0.164)
Logged education level (Year)	−0.381***	−0.045	− 0.426*
	(0.066)	(0.093)	(0.079)
Logged government health expenditure (RMB)	0.210***	−0.046	0.164***
	(−0.045)	(0.061)	(0.060)
Urbanization level (%)	0.745***	0.243	0.988
	(−0.485)	(1.090)	(1.156)
ρ/λ	0.453**		
R-sq	0.963		
Log-likelihood	375.614		

Note: 1 Stand-error shown in parentheses, ***, ** and * indicate significance at the 1, 5 and 10% levels, respectively

2 Per capita OPP, Per capita income, GDP, Education level and Government health expenditure take natural logarithm forms

Concerning the direct effect, among the factors associated with demand, the effects of mortality rate and the ratio of visits in hospitals and those in primary institutions on per capita OPPs were found to be significantly positive, with regression coefficients of0.156 and 0.013, respectively. The calculated per capita income elasticity on per capita OPPs is 0.398. The influence of the level of aging is not significant. Among supply-side factors, the share of primary health beds on residents’ OPPs can be seen to have a negative significant correlation, with a regression coefficient of 0.09. The ratio of health technicians in hospitals to those in primary care and maternal mortality rate have insignificant direct effects on OPPs. Among the socio-economic factors, the direct effects of GDP and government health expenditure on per capita OPPs emerged as positive, with respective elasticity coefficients of 0.05and 0.210, and urbanization has a positive direct effect.

Regarding the indirect effects, among the demand-side factors, the indirect regression coefficients of the aging level, mortality rate, the ratio of visits to hospitals and those to primary institutions, and the per capita income on local OPPs are 0.616, 0.353, 0.034, and 0.206, respectively. On the supply side, the share of primary healthcare beds, and the indirect effects of the ratio of health technicians between hospitals and primary health institutions on per capita OPPs are significantly negative, with regression coefficients of-0.401 and − 0.014, respectively. The indirect effect of maternal mortality is not significant. There are no significant indirect effects on OPPs by socio-economic factors.

## Discussion

The goal of this paper was not only to verify the existence of the spatial clustering of China’s per capita OPPs but also to investigate the main factors affecting OPPs’ variability and itsspatial spillover sources, using a panel data set covering 31 provinces in Chinafrom2005–2016. Thisis vitally important to the formulation of effective, strategic, and health service policies to promote health equity across China’s healthcare system.

The global and local spatial autocorrelation tests identified that per capita OPPs in China manifest clear spatial clustering across provinces, due to the inequity of healthcare-seeking behaviors and differences in the distribution of health resources. The outstanding phenomenon that the spatial correlation levels first increase and then decrease demonstrates that while China’s healthcare reform has achieved initial results in promoting health equity, China’s per capita OPPs’ clusters have gradually eased in recent years. The LISA distribution maps report that the northeastern OPPs are mainly concentrated in the high-high group, which may be due to their similar healthcare-seeking behaviors induced by the correspondence of socio-economic factors and prevalent endemic diseases. In China’s west region, with its underdeveloped economy and educational level, as well as poor natural environment conditions, the low utilization efficiency of healthcare services increased the spatial correlation of per capita OPPs. The fact that human health and financial health resources are more concentrated in China’s richer eastern and central areas, where residents’ demands can be satisfied through the local health system supply, can explain the non-significant spatial cluster in China’s east and central provinces [36].

In order to explore the direct influencing factors of per capita OPPs and the source of spatial spillover, an SDM was constructed for this study. The main findings pertaining to the direct and indirect effects are as follows.

Among the demand-side factors, per capita income, mortality rate, and the ratio of visits between hospitals and primary healthcare facilities were found to have a positive correlation with per capita OPPs. The per capita income directly determines individual residents’ or families’ ability to pay for healthcare services. Si [37] has indicated that poorer people are likely to engage in much lower health expenditure than richer people with the same social status. Groups with a poorer health status have higher OPP spending and bear a heavier economic burden, which can affect their possibilities to seek healthcare. The Chinese government should pool the economic risks through the reimbursement of the health insurance system, and increase subsidies for patients to promote health equity. The positive direct effect of the ratio of visits to hospitals and those to primary institutions suggests that more visits to hospitals or fewer visits to primary healthcare institutions are likely to encourage the increase of OPPs. In China, a number of residents are willing to go to hospitals to seek healthcare services rather than primary healthcare institutions, so shifting this healthcare-seeking model would be essential, and made possible by a series of reforms [38, 39].

In terms of the supply side, the negative direct effect of the share of primary healthcare beds shows that a higher proportion of grassroots beds can control the rapid growth of health costs. Over the past twenty years, the government’s subsidy has mainly concentrated on public hospitals, which has not only given rise to excess resources in these hospitals but has also stimulated the rapid growth of OPPs. The construction of grassroots beds could thus not only improve the fairness of China’s health service distribution but could also relieve the pressure placed on hospitals. Orzol [40] has indicated that patient-centered and community-involved primary healthcare services can help to solve the problem of fragmented supply and the rapid growth of OPPs. Hospitals can make better use of health resources in order to specialize in complex treatment and improve their technology level, thus meeting the higher demands for their health services and, ultimately, promoting the realization of vertical health equity.

Socio-economic factors were found to play the most important role in terms of the direct effects on per capita OPPs. GDP, government health expenditure, and urbanization level were seen to have significant positive direct effects on per capita OPPs. Alongside social and economic development and the living standards of Chinese residents have gradually enhanced, which has promoted the demand for better health services and induced the growth of OPPs. A higher level of urbanization, more stable and sustained economic growth and higher government input are the macro conditions that enable pooling of OPPs’ risk and ensure the sustainability of health financing [41].

Regarding the results of the indirect effects, per capita income, aging level, mortality rates, and the ratio of visits to hospitals and those to primary institutions were found to have significant positive indirect effects on per capita OPPs, demonstrating that the demand-side factors comprise the main, positive spatial OPPs’ spillover source. The improvement of residents’ purchasing power has meant a change in residents’ demand for health services. When people who need higher levels of healthcare are aware that local health services cannot meet their needs, they will seek out better services across different areas, or even choose an area with a higher quality of health services in which to settle. The regions with better healthcare services will increase their prices in order to pursue higher profits and relieve the pressure caused by the limited health service capacity and the rigid demand for high-quality health services, meaning that local per capita OPPs will increase. This also reflects the imbalance between the supply and demand of health services in China (i.e., the supply level cannot meet the needs of local residents).

The negative indirect effects of supply-side factors indicate that the strengthening of human and material resources in primary healthcare services can alleviate the positive spillover effect of per capita OPPs. Healthcare services include basic medical services based on primary medical institutions and complex medical services based on hospitals. High quality and low-cost primary healthcare services that meet residents’ expectations can divert pressure away from hospital diagnosis and treatment, meaning that the number of potential hospital customers will be reduced and the market competition among hospitals intensified. In China, some health service prices regulated by the National Price Commission are lower than costs, and public hospitals assume sole responsibility for their profits or losses [39]. It is the primary responsibility of hospitals to undertake the healthcare services relating to more complex treatments for local residents. Hospitals tend to strengthen technological innovation and service quality to attract local customers and enhance their competitiveness for the purpose of survival, which has the advantage of providing higher quality and cheaper health services for local residents [41]. When residents’ pursuit for higher quality is satisfied, the likelihood of healthcare-seeking behaviors across regions will decrease, which could promote the utilization of health services and achieve universal health coverage. Overall, it is essential to strengthen the labor capacity, material resources and technology of China’s primary healthcare institutions, and enhance residents’ trust in primary healthcare services.

## Conclusion

Given this study’s analysis, the following conclusions can be drawn. Firstly, the spatial correlation test proves that the spatial clustering of per capita OPPs across regions in China still exists, despite the implementation of a series of measures to promote health equity. This indicates an unfair distribution of health resources and utilization of health services in China, especially in the country’s northeast and western regions. Arresting the spatial correlation of per capita OPPs and achieving health equity in China’s health service system thus remains a major challenge. Secondly, our findings show that socio-economic factors and those associated with demand are the main influencing factors of OPPs’ variation, while demand is also the driver of the positive spatial spillover of per capita OPPs, whereby an effective supply could relieve the spatial spillover. With China’s social and economic development, demand has become the dominant factor behind OPPs’ growth. However, regional imbalances between supply and demand can induce the spatial spillover of per capita OPPs. Strengthening the infrastructure and construction of primary healthcare services, and improving the competitiveness of hospitals, can reduce the expense of local per capita OPPs as well as potentially alleviating the OPPs’ spatial spillover. This implication, which emerged from the current study, could provide policy directions for the design of health equity in China.

Based on the findings of this paper, some recommendations can be made for the control of China’s per capita OPPs and for curbing its spatial spillover effects.

Firstly, the quality of primary healthcare services should be enhanced, hospitals’ technological level improved, and health resources integrated. For a long time, the low quality of primary healthcare services has been a key reason why residents have chosen hospitals as their primary choice for medical treatment, instead of cheaper primary healthcare institutions. In order to meet residents’ demand for high quality in the remit of common medical and healthcare services, primary healthcare institutions should establish a vertical cooperation mechanism with large hospitals, ensure the supply of human resources, and enhance their technical capacity under the guidance of hospitals. Hospitals in provinces with a higher level of medical technology should build cross-provincial horizontal cooperation with those in regions with a poorer level. Using modern technology (such as telemedicine) together with cross-regional guidance, patients with complex diseases could be provided with a diagnosis and treatment within their region, which could solve the problem of cross-regional healthcare-seeking caused by China’s uneven distribution of resources.

Secondly, China’s social health insurance system should be reformed. Increasing the reimbursement ratio of outpatients in primary healthcare institutions to hospital inpatients could shift the utilization away from hospital outpatient departments to grassroots beds, and pool the risks of exorbitant health expenditure. Alongside this, the system should provide health insurance packages stipulating the appropriate level of the health institutions and the referral procedures to hospitals in other provinces.

Thirdly, methods of payment and price regulation should be reformed. Some pilot regions have introduced alternative provider payment methods such as DRGs-PPS, which has effectively curbed the provision of unnecessary services and reduced residents’ OPPs. In addition, hospitals should be given the appropriate rights to adjust prices according to residents’ income levels.

Lastly, effective monitoring and management systems should be built. The ratio of local outpatients and inpatients to non-native patients at a healthcare institution should be taken as a performance indicator, as this is conducive to knowing the main diseases, numbers, and expenditure of patients seeking cross-provincial health services. Otherwise, the implementation of health service systems will remain nothing but empty words without the effective supervision of the government.

The current study has some limitations. Firstly, the data from the statistical yearbook could only be accessed at province level and could not be extended to county level, meaning that it was not possible to analyze the internal spatial spillover effect for a single province. Secondly, while the spatial effects and spillover sources of OPPs were measured, this study did not identify the spatial flow pattern of particular components (hospitalization, physicians, nursing care, and prevention, etc.).Thirdly, PHE, SHE, and OPPs jointly promote the healthy circulation of healthcare activities and health equity. So, the sources of the spatial spillover of GHE and SHE are still to be studied. These remain important issues to be addressed in future research.

## Additional file

Additional file 1: **Table S1.** Composition of China’s Health Expenditure in 2016. (DOCX 22 kb)

## Data Availability

This was a secondary analysis. All data can be obtained from http://data.stats.gov.cn/easyquery.htm?cn=E0103 and http://www.nhc.gov.cn/wjw/index.shtml. To request this data, please contact to Xia Fang (Email: Xiafang425@126.com).

## Abbreviations

DRGs-PPS: Diagnosis Related Groups-Prospective Payment System
ESDA: Exploratory Spatial Data Analysis
GDP: Gross Domestic Product
GHE: Government Health Expenditure
LISA: Local Indicators of Spatial Associations
NCME: New Rural Cooperative Medical Insurance
OPPs: Out-of-Pocket Payments
PHE: Public Health Expenditure
SDM: Spatial Durbin Model
SHE: Social Health Expenditure
THE: Total Health Expenditure
UEMI: Urban Employees Medical Insurance
URMI: Urban Residents Medical Insurance
