# Supplementary material for: What has driven the spatial spillover of China’s out-of-pocket payments?
Source: BMC Health Serv Res. 2019 Aug 30;19:610. doi: 10.1186/s12913-019-4451-0 (PMC6716932; doi:10.1186/s12913-019-4451-0)
Supplement: Supplementary file 1 — Table S1. Composition of China’s Health Expenditure in 2016. (DOCX 22 kb) [file 12913_2019_4451_MOESM1_ESM.docx]

**Additional file 1: TableS1. Composition of China's Health Expenditure in 2016**

| Regions | Provinces | Health expenditure（Billon RMB） | | | | Proportion (%) | | | Per capita Income（RMB） |
| --- | --- | --- | --- | --- | --- | --- | --- | --- | --- |
|  |  | THE | GHE | SHE | OPP | GHE | SHE | OPP |  |
| East | Beijing | 2048.99 | 467.99 | 1247.23 | 333.76 | 22.84 | 60.87 | 16.29 | 52530.38 |
|  | Tianjin | 827.02 | 211.76 | 361.64 | 253.62 | 25.60 | 43.73 | 30.67 | 34074.46 |
|  | Hebei | 2024.82 | 567.79 | 720.65 | 736.38 | 28.04 | 35.59 | 36.37 | 19725.42 |
|  | Liaoning | 1484.46 | 316.59 | 626.64 | 541.24 | 21.33 | 42.21 | 36.46 | 26039.70 |
|  | Shanghai | 1838.00 | 430.73 | 1061.73 | 345.53 | 23.43 | 57.77 | 18.80 | 54305.35 |
|  | Jiangsu | 3359.58 | 753.25 | 1754.96 | 851.37 | 22.42 | 52.24 | 25.34 | 32070.10 |
|  | Zhejiang | 2573.55 | 557.81 | 1295.37 | 720.36 | 21.67 | 50.33 | 27.99 | 38529.00 |
|  | Fujian | 1250.07 | 388.46 | 547.97 | 313.64 | 31.08 | 43.84 | 25.09 | 27607.93 |
|  | Shandong | 3354.70 | 813.19 | 1536.92 | 1004.59 | 24.24 | 45.81 | 29.95 | 24685.27 |
|  | Guangdong | 4193.33 | 1158.66 | 2009.16 | 1025.50 | 27.63 | 47.91 | 24.46 | 30295.80 |
|  | Hainan | 303.28 | 116.54 | 118.75 | 67.99 | 38.43 | 39.16 | 22.42 | 20653.44 |
| Center | Shanxi | 975.76 | 309.73 | 363.03 | 303.00 | 31.74 | 37.20 | 31.05 | 19048.88 |
|  | Jilin | 956.89 | 280.02 | 352.48 | 324.39 | 29.26 | 36.84 | 33.90 | 19966.99 |
|  | Heilongjiang | 1190.45 | 287.45 | 486.94 | 416.06 | 24.15 | 40.90 | 34.95 | 19838.50 |
|  | Anhui | 1643.29 | 550.55 | 597.92 | 494.82 | 33.50 | 36.39 | 30.11 | 19998.10 |
|  | Jiangxi | 1090.56 | 468.18 | 325.37 | 297.02 | 42.93 | 29.84 | 27.24 | 20109.56 |
|  | Henan | 2472.63 | 794.42 | 859.06 | 819.15 | 32.13 | 34.74 | 33.13 | 18443.08 |
|  | Hubei | 1924.72 | 611.23 | 669.42 | 644.07 | 31.76 | 34.78 | 33.46 | 21786.64 |
|  | Hunan | 1924.47 | 560.26 | 743.41 | 620.80 | 29.11 | 38.63 | 32.26 | 21114.79 |
| West | Inner Mongolia | 907.16 | 303.94 | 299.70 | 303.52 | 33.50 | 33.04 | 33.46 | 24126.64 |
|  | Guangxi | 1237.09 | 475.69 | 437.50 | 323.90 | 38.45 | 35.37 | 26.18 | 18305.08 |
|  | Chongqing | 1064.57 | 339.75 | 411.82 | 312.99 | 31.91 | 38.68 | 29.40 | 22034.14 |
|  | Sichuan | 2675.77 | 784.42 | 1111.62 | 779.72 | 29.32 | 41.54 | 29.14 | 18808.26 |
|  | Guizhou | 878.93 | 403.29 | 270.34 | 205.30 | 45.88 | 30.76 | 23.36 | 15121.15 |
|  | Yunnan | 1313.85 | 473.34 | 463.17 | 377.35 | 36.03 | 35.25 | 28.72 | 16719.90 |
|  | Xizang | 124.98 | 85.54 | 32.60 | 6.83 | 68.45 | 26.09 | 5.46 | 13639.24 |
|  | Shaanxi | 1348.15 | 391.70 | 527.58 | 428.87 | 29.05 | 39.13 | 31.81 | 18873.74 |
|  | Gansu | 754.06 | 283.95 | 250.60 | 219.51 | 37.66 | 33.23 | 29.11 | 14670.31 |
|  | Qinghai | 239.75 | 111.83 | 61.95 | 65.98 | 46.64 | 25.84 | 27.52 | 17301.76 |
|  | Ningxia | 251.77 | 85.59 | 93.10 | 73.09 | 33.99 | 36.98 | 29.03 | 18832.28 |
|  | Xinjiang | 962.32 | 299.05 | 415.58 | 247.70 | 31.08 | 43.18 | 25.74 | 18354.65 |
| Average | East | 2114.35 | 525.71 | 1025.55 | 563.09 | 26.06 | 47.22 | 26.72 | 32774.26 |
|  | Center | 1522.35 | 482.73 | 549.70 | 489.91 | 31.82 | 35.82 | 32.01 | 20038.32 |
|  | West | 979.87 | 336.51 | 364.63 | 278.73 | 38.50 | 34.92 | 26.58 | 18065.60 |

Notes: THE, GHE, SHE, OPP stand for total health expenditure, government health expenditure, social health expenditure and out-of-pocket payment, respectively.
